# Combination of antibody–drug conjugates with immunotherapy: a new paradigm in cancer treatment

**DOI:** 10.3389/fonc.2026.1945452

**Published:** 2026-10-22

**Authors:** Yongli Lai, Ruyu Qin, Zhaidong Liu

**Affiliations:** 1 The First Clinical Medical College, Shandong University of Traditional Chinese Medicine, Jinan, China; 2 School of Traditional Chinese Medicine, Shandong Second Medical University, Weifang, China; 3 Department of Oncology, Affiliated Hospital of Shandong University of Traditional Chinese Medicine, Jinan, China

**Keywords:** antibody-drug conjugates, combination therapy, immunotherapy, synergistic mechanisms, tumor microenvironment

## Abstract

Immunotherapy, which activates the host immune system to recognize and eliminate tumor cells, has become an important modality in cancer treatments. However, tumor heterogeneity and the immunosuppressive tumor microenvironment often drive resistance to single-agent immunotherapy, thereby limiting its clinical efficacy. Antibody–drug conjugates (ADCs) are a class of targeted anticancer agents composed of a monoclonal antibody, a linker, and a cytotoxic payload. In addition to direct tumor cell killing, ADCs can remodel the tumor microenvironment through multiple mechanisms, thereby contributing to the reversal of immune resistance. Consequently, combination strategies integrating ADCs with immunotherapy have attracted increasing attention. Clinical studies have demonstrated encouraging benefits of ADC–immune checkpoint inhibitor combinations across multiple tumor types, and several emerging regimens have shown promising therapeutic potential. Nevertheless, significant challenges remain in clinical translation. This review summarizes the synergistic mechanisms and recent advances in ADC–immunotherapy combinations, discusses current limitations, and outlines future directions, with the aim of guiding the precise and standardized development of these combination strategies.

## 1 Introduction

Malignant tumors remain a major public health threat to human life and well-being, accounting for approximately 10 million deaths annually worldwide, with the global annual incidence projected to reach 30 million by 2040 (1). In the face of this escalating disease burden, traditional treatment strategies are no longer sufficient to meet clinical needs, and there is an urgent need to explore new treatment approaches.

Immunotherapy has become an important treatment modality for various malignancies by overcoming tumor-induced immune tolerance, restoring the function of exhausted T cells, and enhancing antitumor immune responses (2, 3). Immune checkpoint blockade therapy, as represented by immune checkpoint inhibitors (ICIs), has significantly improved patient survival outcomes in various solid tumors; among these, programmed cell death protein 1 (PD-1)/programmed death-ligand 1 (PD-L1) pathway blockade is currently the treatment strategy with the strongest clinical evidence (4). In addition, strategies such as cell therapy, tumor vaccines, and immune agonists can activate the immune system through mechanisms independent of immune checkpoints, representing another emerging direction in immune regulation (5, 6). However, responses to immunotherapy vary substantially among patients; some patients fail to derive clinical benefit, whereas others develop acquired resistance, limiting the overall clinical efficacy of immunotherapy (7).

As a key representative of precision-targeted therapy, antibody-drug conjugates (ADCs) achieve tumor-specific cytotoxicity through a tripartite structure comprising a targeting antibody, a linker, and a cytotoxic payload (8). Although ADCs as monotherapies have demonstrated significant antitumor activity in various solid tumors, the single-target killing mechanism has inherent limitations; antigen heterogeneity, antigen downregulation, and the evolution of drug resistance can all limit their ability to achieve long-term disease control (9). Therefore, overcoming the limitations of ADC monotherapy has become a major research focus in the field of precision oncology.

Recent studies have shown that a complex bidirectional regulatory relationship exists between ADCs and the tumor microenvironment (TME) (10). On the one hand, ADCs can remodel the tumor immune microenvironment through multiple pathways, transforming immunosuppressive “cold tumors” into immune-responsive “hot tumors”; on the other hand, the tumor immune microenvironment can also reciprocally regulate the intratumoral delivery, accumulation, and efficacy of ADCs (11, 12). It should be noted that the regulatory effect of ADCs on the immune microenvironment is bidirectional, and the bystander effect and nonspecific exposure to free payloads may impair the function of neighboring immune cells and other normal cells (13). Therefore, the dynamic interaction between ADCs and the TME provides an important theoretical foundation for ADC-immunotherapy combinations, but the overall antitumor efficacy of these combinations is context-dependent. Currently, the combination of ADCs with PD-1/PD-L1 inhibitors has the strongest clinical evidence: ADC-induced tumor antigen release and immune activation can enhance the effects of PD-1/PD-L1 blockade, while PD-1/PD-L1 inhibitors can further amplify the ADC-mediated antitumor immune response (14). Meanwhile, emerging strategies such as the combination of ADCs with immune agonists or cell therapies, as well as immunostimulatory dual-payload ADCs have further expanded the range of combination regimens (15).

Overall, ADC-immunotherapy combinations represent an important approach to overcoming the limitations of monotherapy and may provide new treatment options for patients with malignant tumors. However, these combination strategies still face many unresolved issues in clinical practice. This review aims to outline the mechanistic basis of ADC-immunotherapy combinations, summarize the current clinical research progress of ADC–ICI combination regimens with the most robust clinical evidence to date, further discuss emerging immunotherapeutic combination strategies, and address the major challenges and future directions, with the goal of providing a reference for related basic research and clinical translation.

## 2 Literature search

To ensure a systematic and reproducible review, a standardized literature search and screening strategy was implemented. The literature search was conducted using PubMed, Web of Science, and other relevant databases. Search terms included “antibody–drug conjugates”, “immunotherapy”, “tumor microenvironment”, “combination therapy”, and “synergistic mechanisms”. The search covered the period from database inception to June 2026.

The inclusion criteria were as follows: (1) basic, translational, or clinical studies directly investigating the combination of ADCs and immunotherapy; (2) high-quality review articles or meta-analyses; and (3) opinion articles or commentaries exploring the therapeutic potential of this combination. The exclusion criteria included conference abstracts, case reports, and studies with incomplete data.

The literature screening process was conducted independently by two researchers. Titles and abstracts were initially screened, followed by a full-text review of studies that met the eligibility criteria. Any disagreements between the two researchers were resolved through discussion or consultation with a third expert in the field.

## 3 Mechanisms of combination therapy

ADCs and the TME together form a dynamic, bidirectional interactive network that collectively influences the efficacy of ADC-immunotherapy combinations. On the one hand, ADCs multidimensionally reshape the tumor immune microenvironment and activate antitumor immune responses through payload-mediated immunogenic cell death (ICD), Fc-driven innate immune activation, and the cleavable linker-mediated bystander effect. However, the diffusion and systemic exposure of payloads may also damage immune cells. On the other hand, the degree of immune infiltration, microenvironmental barriers, and inhibitory signaling pathways in the TME can modulate the therapeutic efficacy of ADCs, but generally do not affect the direct cytotoxic effect of the ADC payload on tumor cells. This chapter elucidates the theoretical basis for ADC-immunotherapy combinations by detailing the bidirectional interaction mechanisms between ADCs and the tumor immune microenvironment.

### 3.1 Regulatory effects of ADCs on the immune system

The immunomodulatory effects of ADCs arise from multifaceted immune remodeling driven by the coordinated actions of their three components—monoclonal antibodies, linkers, and cytotoxic payloads. These actions not only link innate and adaptive immunity but also provide the mechanistic basis for the synergistic effects when ADCs are combined with immunotherapy. **Figure 1** illustrates how the three components remodel the tumor immune microenvironment.

**Figure 1:**
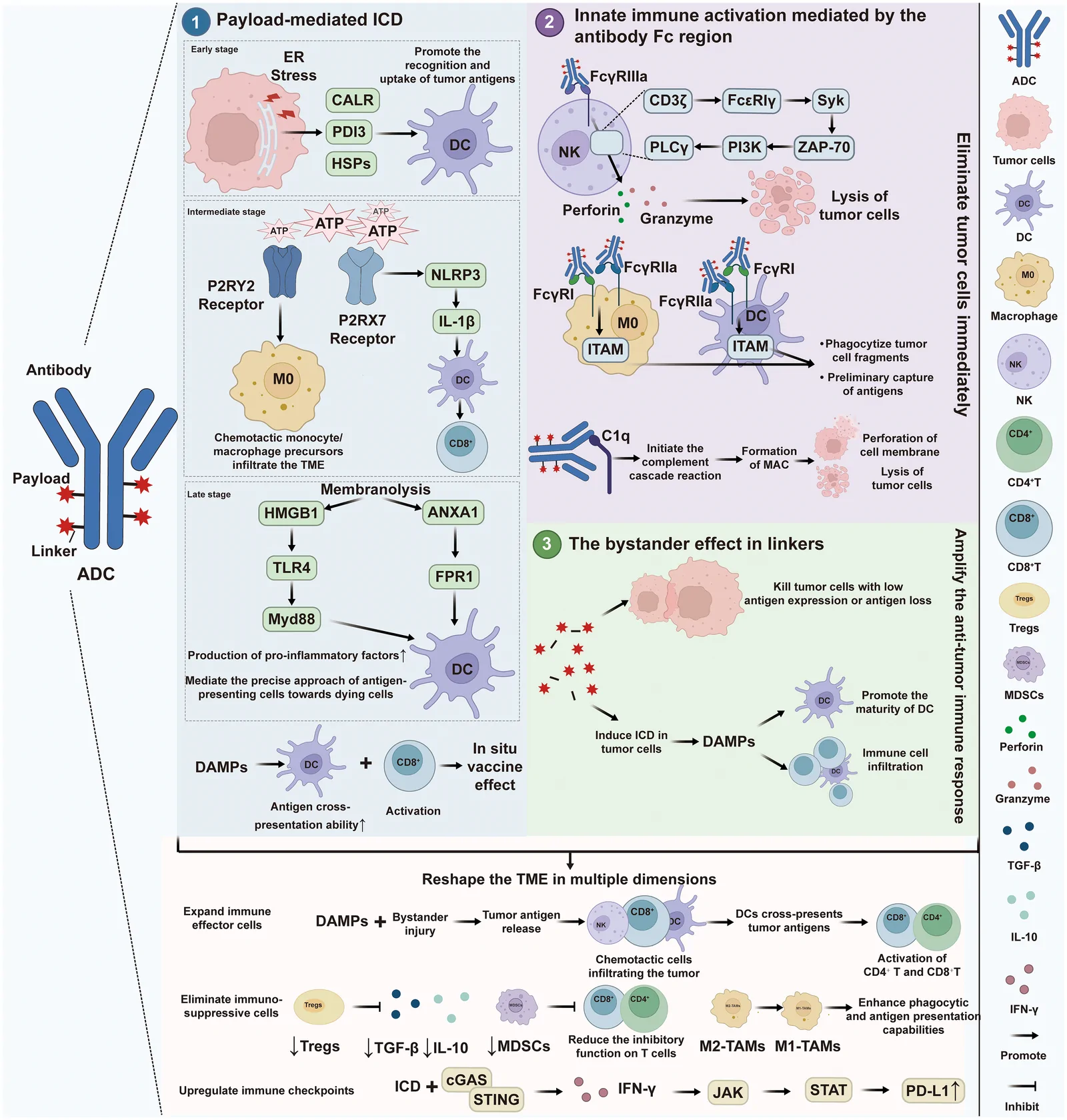
Schematic diagram of the multi-layer immunoregulatory mechanisms of ADCs. The figure illustrates the complete process by which three core pathways—payload-mediated ICD, Fc-driven innate immune activation, and bystander effects mediated by linkers—remodel the tumor immune microenvironment. The blue Y-shaped structure represents ADC; pink cells represent tumor cells, purple cells represent DCs, beige cells represent macrophages, pale gray-purple cells represent NK cells, light green cells represent CD4^+^ T cells, light blue cells represent CD8^+^ T cells, pale yellow cells represent Tregs, and lavender cells represent MDSCs. Among the scattered signal dots, green dots represent perforin, red dots represent granzyme, and dark blue, light cyan, and pale pink dots represent TGF-β, IL-10, and IFN-γ, respectively. → indicates promotion/activation, and ⊣ indicates inhibition/blockade.

#### 3.1.1 Payload-mediated immunogenic cell death

The antitumor activity of ADCs is derived not only from the direct cytotoxic effects of the payload on tumor cells but also, more importantly, from the induction of ICD. ICD is a distinct form of regulated cell death characterized by the orderly exposure and release of a series of damage-associated molecular patterns (DAMPs) (16, 17). ADC payloads trigger endoplasmic reticulum stress and autophagy in tumor cells through intracellular mechanisms, thereby initiating the DAMP release cascade associated with ICD (18, 19). Early endoplasmic reticulum stress induces the translocation of molecules such as calreticulin, protein disulfide isomerase A3 (PDIA3), and heat shock proteins to the cell surface, thereby promoting the recognition and uptake of tumor cell antigens by dendritic cells (DCs) (20–22). During the intermediate phase, large amounts of intracellular ATP are released into the extracellular space; on the one hand, this recruits monocyte/macrophage precursors via the P2RY2 receptor into the TME (23), and on the other hand, it acts on the P2RX7 receptor expressed on DCs, activating the NLRP3 inflammasome and promoting interleukin-1β (IL-1β) maturation and secretion, thereby driving DC maturation and subsequent CD4^+^/CD8^+^ T-cell responses (24). In the late stage, as cell membrane integrity is lost, high-mobility group box 1 (HMGB1) and annexin A1 (ANXA1) are released into the extracellular space. The former promotes DC maturation and pro-inflammatory cytokine production through the TLR4–MyD88 signaling pathway (25), whereas the latter binds to formyl peptide receptor 1 expressed on DCs, mediating the precise homing of antigen-presenting cells to dying cells (26). These danger signals synergistically enhance the antigen cross-presentation capacity of DCs, activating CD4^+^ T cells and CD8^+^ T cells, thereby generating an *in situ* vaccine effect at the tumor site and establishing long-lasting antitumor immune memory. It is worth noting that the molecular pathways through which different ADC payloads induce ICD vary. Microtubule-targeting payloads primarily trigger DAMP release by activating the endoplasmic reticulum stress pathway (27), whereas DNA-damaging payloads can also trigger a type I interferon (IFN-I) response through the cytoplasmic DNA-sensing pathway, resulting in dual immune activation (28). This heterogeneity suggests that the selection of ADC payloads not only affects direct cytotoxic efficacy but also shapes their immunomodulatory profiles. Therefore, the design of combination therapies should take into account the distinct mechanisms of ICD induction associated with different ADC payloads.

#### 3.1.2 Innate immune activation mediated by the antibody Fc domain

Most immunoglobulin G (IgG)-type ADCs retain the Fc domain. Upon specific binding of the Fab domain to target antigens on the tumor cell surface, the ADC becomes anchored to the target cell membrane. This interaction brings its Fc domain into proximity with Fcγ receptors (FcγRs) expressed on adjacent immune effector cells or with serum complement component 1q (C1q), thereby initiating multiple innate immune activation pathways that are independent of payload internalization (12, 29). (1) Antibody-dependent cellular cytotoxicity (ADCC): Upon binding of FcγRIIIa, which is highly expressed on natural killer (NK) cells, to the Fc domain of an ADC, the associated CD3ζ and FcϵRIγ signaling chains activate the Syk/ZAP-70/PI3K/PLCγ signaling cascade, stimulating NK cells to release cytotoxic mediators such as perforin and granzyme B. These mediators penetrate the tumor cell membrane, inducing target cell lysis and apoptosis (30, 31). (2) Antibody-dependent cellular phagocytosis (ADCP): Macrophages and DCs recognize and bind to the Fc domain of ADCs through the activating receptors FcγRI and FcγRIIa, triggering immunoreceptor tyrosine-based activation motif (ITAM)-mediated phagocytosis of tumor cell debris (32–34). Simultaneously, they capture tumor-associated antigens, facilitating subsequent antigen presentation and activation of adaptive immune responses (35). (3) Complement-dependent cytotoxicity (CDC): The Fc domain binds to C1q in serum, initiating the classical complement cascade, which ultimately leads to the formation of the membrane attack complex (MAC) on the tumor cell surface, causing membrane disruption and complement-mediated tumor cell lysis (36–38). Through Fc-mediated activation of multiple innate immune effector functions, ADCs rapidly remodel the TME, promoting immediate tumor cell clearance and enhanced antigen presentation, thereby laying the groundwork for subsequent adaptive immune responses.

#### 3.1.3 The cleavable linker-mediated bystander effect

Linkers are critical for enabling stable conjugation between the antibody and the cytotoxic payload in ADCs and are broadly classified as either cleavable or non-cleavable. Their cleavage properties largely determine the magnitude of the bystander effect, an important structural feature that enhances local immune responses and facilitates the elimination of antigen-negative tumor cells. After an ADC binds to tumor antigens via its Fab domain and is internalized into lysosomes, the cleavable linker is cleaved by lysosomal acidic proteases, releasing its cytotoxic payload. A proportion of the released payload is not restricted to the target cells in which the ADC was internalized but instead diffuses across cell membranes into adjacent tumor cells, stromal cells, and immune cells, thereby exerting a bystander effect (39). On the one hand, the diffused payload can eliminate tumor cells with low antigen expression or antigen loss, compensating for the limitation that ADCs primarily target antigen-positive tumor cells (40). On the other hand, payload diffusing into the TME can simultaneously induce ICD in neighboring tumor cells, leading to the release of additional DAMPs, further promoting immune cell infiltration and DC maturation, and continuously amplifying the *in situ* vaccine effect (28). In contrast, non-cleavable linkers are degraded only after target cell apoptosis, together with the resulting cellular debris. Consequently, the released payload exhibits minimal diffusion capacity and little to no bystander effect (41). However, the “bystander effect” is not entirely beneficial for antitumor immunity; the released free payload may also impair the survival and effector functions of immune cells in the microenvironment (42). In summary, the bystander effect mediated by cleavable linkers is dual in nature, involving both immune activation and immune suppression: while it can enhance antitumor immunity by eliminating antigenically heterogeneous tumor cells and expanding the scope of ICD induction, the diffusion of the payload to non-tumor cells may also weaken the local immune response. Its immunological effects relate to the type of payload, the stability of the linker, and the characteristics of the local microenvironment.

#### 3.1.4 ADC-driven remodeling of the tumor immune microenvironment

Through the synergistic action of three key components—antibody, payload, and linker—ADCs comprehensively reprogram the TME at three levels: immune effector cells, immunosuppressive cells, and tumor cell–intrinsic molecular expression. First, ADCs induce the release of large amounts of DAMPs and trigger bystander killing, continuously releasing tumor-associated antigens, which recruit CD8^+^ T cells, NK cells, and mature DCs from the peripheral circulation to infiltrate the tumor site (28). Mature DCs cross-present captured tumor antigens, thereby promoting antigen-specific activation of CD4^+^ T cells and CD8^+^ T cells (43, 44). Second, when the target antigen is highly expressed on the surface of regulatory T cells (Tregs), ADCs can eliminate some Tregs through targeted action, thereby reducing the secretion of immunosuppressive factors such as interleukin-10 (IL-10) and transforming growth factor beta (TGF-β) (45, 46); simultaneously, ADCs can also inhibit the proliferation and differentiation of myeloid-derived suppressor cells (MDSCs), reducing their inhibitory effects on T cells (47, 48), and promote the polarization of M2-polarized tumor-associated macrophages (TAMs) toward the antitumor M1 phenotype, thereby enhancing local phagocytosis and antigen presentation capabilities (49). Furthermore, ADCs can induce ICD and activate the cyclic GMP-AMP synthase–stimulator of interferon genes (cGAS–STING) pathway, resulting in the release of IFN-I, which in turn upregulates PD-L1 expression via the JAK–STAT signaling axis, providing a therapeutic target for ICIs (50, 51). In summary, ADCs reshape the TME through multiple dimensions: they expand effector immune cell populations, reduce multiple immunosuppressive signals, and upregulate immune checkpoint molecules on tumor cells. These effects establish the molecular foundation at the microenvironmental level for the enhanced efficacy of ADC–immunotherapy combinations.

It is worth noting that the effects of ADCs on the immune system are not limited to the immune remodeling mediated by the three components mentioned above; they can also create favorable conditions for immune cells to infiltrate tumor sites by directly disrupting tumor tissue structure and weakening local tissue barriers. The sustained death of tumor cells and surrounding tissues induced by ADCs can also reduce hydrostatic pressure in the tumor stroma and lessen the physical barrier posed by the fibrotic matrix to immune cells, further promoting the penetration of effector immune cells into the deep layers of the tumor (52, 53). However, systemic exposure to ADCs and their payloads may also exert nonspecific toxicity on circulating immune cells as well as those in the bone marrow and other organs, leading to a reduction in the number of peripheral immune cells, altered activation states, and impaired function, which in turn affects their ability to infiltrate tumors and their local immune phenotype (13, 54). Therefore, ADC-induced local immune activation may coexist with potential immune cell damage, and combination therapy strategies must fully consider the balance between the two.

### 3.2 Impact of immune status on ADC efficacy

#### 3.2.1 Impact of immune cell infiltration status on ADC therapeutic efficacy

The degree of immune cell infiltration directly influences the antitumor response of ADCs, and the number of tumor-infiltrating lymphocytes (TILs) is a key determining factor (55). In “hot tumors” with high infiltration of effector T cells (Teffs), ADCs can induce ICD to release DAMPs via their payload, thereby driving DC maturation and cross-presentation of tumor antigens (56); simultaneously, the Fc domain of IgG-type ADCs can mediate innate immune effects such as ADCC in preclinical models (57), but its contribution to the overall clinical efficacy of ADCs remains unclear. Studies have shown that “bicycle drug conjugates” (BDCs), which lack an Fc domain, exhibit antitumor efficacy in clinical settings similar to that of ADCs carrying the same payload, suggesting that the clinical benefits of ADCs may not primarily depend on Fc-mediated ADCC (58). In preclinical studies, the combination of Fc-silenced conjugates with PD-1/PD-L1 inhibitors has also demonstrated favorable combined antitumor activity, further indicating that even in the absence of Fc-mediated ADCC, conjugate-induced ICD and immune activation can still contribute to the efficacy of combination therapy (59). Pre-existing CD8^+^ T cells within the TME recognize major histocompatibility complex class I (MHC-I)–presented tumor antigens via their T-cell receptor; upon stimulation by pro-inflammatory cytokines such as interferon alpha (IFN-α) and interleukin-12 (IL-12), they rapidly proliferate and become activated, efficiently eliminating primary tumors and micrometastases. These cells can further differentiate into memory T cells, thereby enabling durable antitumor immunity (60–62). In contrast, in “cold tumors” lacking Teffs, even if ADCs induce ICD to release DAMPs, tumor antigens presented by DCs fail to initiate a complete T-cell activation cascade due to insufficient functional CD8^+^ T cells as downstream effectors of the immune response (63, 64). Moreover, immunosuppressive cytokines such as IL-10 and TGF-β secreted by Tregs further inhibit the proliferation and cytotoxic function of residual T cells, thereby preventing the establishment of systemic antitumor immunity and limiting ADC activity to transient local tumor control (65).

#### 3.2.2 TME barriers limit ADC intratumoral delivery and accumulation

In the TME, abundant collagen fibers and cancer-associated fibroblasts form a dense fibrotic stroma, which—together with elevated interstitial fluid pressure—constitutes a physical barrier that impedes the penetration of macromolecular drugs, limiting efficient diffusion of ADCs from blood vessels into tumor parenchyma and significantly reducing intratumoral ADC accumulation (39, 66). In addition, FcγRs expressed at high levels on M2-polarized TAMs can bind and sequester ADC molecules within the TME (67), thereby reducing the local effective drug concentration and further decreasing the number of functionally active ADCs available for specific binding to tumor target antigens (68). Through these two dimensions—physical barriers and cellular barriers—the TME limits effective ADC delivery to tumors, thereby constraining their antitumor efficacy.

#### 3.2.3 The immunosuppressive microenvironment participates in ADC resistance

The immunosuppressive TME participates in both intrinsic and acquired resistance to ADCs through multiple molecular mechanisms. In intrinsic resistance, tumor-infiltrating immunosuppressive cells constitutively secrete TGF-β and IL-10 (69). TGF-β suppresses NK cell activation and cytotoxic function, thereby impairing Fc-mediated ADCC (70); it also inhibits cGAS–STING signaling in DCs, thereby blocking ICD-associated IFN-I production and antigen presentation (71). IL-10 inhibits macrophage polarization and T-cell activation, thereby impairing both innate and adaptive antitumor immunity (72, 73), reducing the overall efficacy of ADC therapy. It should be noted that the immunosuppressive microenvironment primarily impairs immune-related antitumor effects such as ADCC and ICD, but does not directly block the cytotoxic effects mediated by the ADC payload (74). Therefore, even under immunosuppressive conditions, ADCs can still directly kill tumor cells and other cells exposed to the payload by releasing it; however, their additional immune-mediated antitumor effects will be limited. In acquired resistance, local inflammation induced by ADC-mediated tumor killing paradoxically recruits large numbers of immunosuppressive cells, which gradually accumulate inhibitory mediators (75). Sustained cytokine signaling induces CD8^+^ T-cell exhaustion, characterized by upregulation of inhibitory receptors such as PD-1 and T-cell immunoglobulin and mucin domain-containing protein 3 (TIM-3), resulting in functional impairment of Teffs (76). Concurrently, tumor cells upregulate PD-L1 in a stress-responsive manner, reinforcing immune checkpoint signaling and facilitating immune evasion (77), ultimately leading to reduced long-term ADC efficacy.

Preclinical studies have also provided experimental validation for the combination strategy. In a cervical cancer model, an intercellular adhesion molecule 1 (ICAM1)-targeted ADC depleted tumor-promoting vascular endothelial growth factor A+ (VEGFA^+^) neutrophils via FcγR-mediated phagocytosis, induced IFN-I signaling, increased CD8^+^ T-cell infiltration, and enhanced the efficacy of anti–PD-1 therapy (78). In a mouse model of HER2-positive breast cancer (BC), a novel HER2-targeted ADC promoted CD8^+^ T-cell infiltration by inducing ICD; combination with anti–PD-1 blockade significantly enhanced antitumor efficacy and established long-term immune memory (79). In a triple-negative breast cancer (TNBC) model, a B7 homolog 3 (B7-H3)-targeted dual-payload ADC reshaped the TME by activating cGAS–STING signaling; when used in combination with a PD-L1/4-1BB bispecific antibody, it effectively suppressed tumor recurrence and metastasis (64).

## 4 Clinical research progress on ADC combination therapy with ICI

There is a bidirectional interaction between ADCs and the tumor immune microenvironment, which provides a theoretical basis for the combination of ADCs with ICIs. To date, a substantial body of clinical trial evidence has been accumulated regarding ADC–ICI combination therapy for various solid tumors, and the differing patterns of benefit across cancer types and treatment stages are gradually being elucidated. **Table 1** summarizes representative studies and their key findings.

**Table 1:** Summary of key clinical trials combining ICIs with ADCs.

Cancer type	Trial number	Drugs	Key outcomes	References
UC	NCT04223856	EV + Pembrolizumab vs Gemcitabine + Cisplatin/Carboplatin	mPFS:12.5 vs 6.3mo; mOS:31.5 vs 16.1mo; Consistent survival benefits were observed regardless of cisplatin eligibility status, PD-L1 expression levels, or the presence of liver metastases	80
UC	NCT05302284	RC48 + Toripalimab vs Gemcitabine+Cisplatin/ Carboplatin	mPFS: 13.1 vs 6.5mo; mOS: 31.5 vs 16.9	81
UC	NCT05327530	Avelumab + SG vs Avelumab	mPFS:11.17 vs 3.75mo; ORR: 24.3% vs 2.7%; Combination group grade ≥3 TRAEs occurring in 69.9% of patients	82
MIBC	NCT03924895	EV + Pembrolizumab + Surgery vs Surgery	2-y EFS: 74.7% vs 39.4%; 2-y OS: 79.7% vs 63.1%; pCR: 57.1% vs 8.6%; All combination groups presented adverse events	83
BC	NCT05382286	SG + Pembrolizumab vs Pembrolizumab + Taxanes/Gemcitabine + Carboplatin	mPFS: 11.2 vs 7.8mo; mDOR: 16.5 vs 9.2mo;	84
BC	NCT04448886	SG + Pembrolizumab vs SG	The primary endpoint of PFS was not met. A numerical improvement in PFS and OS was observed in the PD-L1-positive (CPS≥1) subgroup	85
BC	NCT03742102	Durvalumab +T-DXd	ORR did not reach the threshold; mPFS: 12.6mo; mOS: 30.3mo	86
NSCLC	NCT04526691	Dato-DXd + Pembrolizumab vs Dato-DXd+ Pembrolizumab+ Platinum chemotherapy	ORR54.8% vs 55.6%; mPFS 11.2 vs 6.8mo; mOS NR vs 17. 4mo; ≥3TRAEs:37.1%vs 59.7%	87
NSCLC	NCT06448312	Sacituzumab Tirumotecan + Pembrolizumab vs pembrolizumab	mPFS: NR vs 5.7mo; 12-mo PFS rate:62% vs 29%; ORR: 70% vs 42%	88
NSCLC	NCT04609566	BV + Pembrolizumab	The absolute therapeutic efficacy was modest. Exploratory biomarker analyses suggested that BV may remodel the TME and potentially overcome PD-1 resistance by eliminating CD30+ Tregs.	89
GC	NCT04280341	RC48 + Toripalimab	ORR:43%; RP2D subgroup: ORR:50%; mPFS:5.1mo, mOS:14.0mo, Four patients developed treatment-related pneumonitis	90
GC	Real-world retrospective analysis	RC48 + PD-1 inhibitor vs RC48	ORR: 41.7% vs 9.5%; mOS: 13.2 vs 7.1mo;	91
HNSCC	NCT04225117	EV + Pembrolizumab	ORR: 39.0%; CR: 9.8%; mPFS: 5.1mo; Subgroup analysis: 81.8% ORR in p16-positive oropharyngeal carcinoma patients	92
USC	NCT03835819	MIRV + Pembrolizumab	ORR: 28%; PFS6 rate: 24.4%; (Preliminary; trial closed early; n=18 of planned 35)	93

### 4.1 Urothelial carcinoma

Urothelial carcinoma (UC) is the solid tumor in which ADC–ICI combination regimens have been most extensively studied. Currently, this combination therapy has established a tiered evidence base across first-line treatment, maintenance therapy, and perioperative settings; however, its clinical translational value and implementation challenges vary significantly across different treatment contexts.

In the first-line setting, the global multicenter phase III EV-302 study (80) enrolled 886 treatment-naïve patients with locally advanced or metastatic UC, who were randomized 1:1 to receive enfortumab vedotin (EV) plus pembrolizumab or standard platinum-based chemotherapy. With a median follow-up of 17.2 months, the median progression-free survival (mPFS) in the combination group was 12.5 months, significantly superior to 6.3 months in the chemotherapy group (HR = 0.45, P < 0.001); the median overall survival (mOS) reached 31.5 months versus 16.1 months in the chemotherapy group (HR = 0.47, P < 0.001). The overall response rate was 67.7%, and the complete response (CR) rate was 29.1%, compared with 44.4% and 12.5%, respectively, in the chemotherapy group. The incidence of grade ≥3 treatment-related adverse events (TRAEs) in the combination group was 55.9%, lower than the 69.5% observed in the chemotherapy group. Consistent survival benefits were observed regardless of cisplatin eligibility status, PD-L1 expression levels, or the presence of liver metastases. The combination of EV and pembrolizumab has been approved by the FDA. This regimen demonstrates clinical efficacy independent of PD-L1 expression, which may be attributed to the monomethyl auristatin E (MMAE) payload of EV inducing ICD in tumor cells, thereby promoting the release of tumor neoantigens and remodeling the TME. This approach holds promise for reducing the dependence of ICI therapy on the baseline tumor immune status.

Another phase III randomized controlled trial (81) targeting patients with HER2-positive advanced UC enrolled 484 treatment-naïve patients, who were randomized to receive disitamab vedotin (RC48) plus toripalimab or platinum-based chemotherapy. With a median follow-up of 18.2 months, the mPFS in the combination group was 13.1 months versus 6.5 months in the chemotherapy group (HR = 0.36, P < 0.001), and the mOS was 31.5 months versus 16.9 months (HR = 0.54, P < 0.001). The objective response rate (ORR) was 76.1% compared with 50.2% in the chemotherapy group. The incidence of grade ≥3 TRAEs in the combination group was 55.1%, lower than the 86.9% observed in the chemotherapy group. The survival benefit of this regimen is comparable to that of EV plus pembrolizumab, but it is more suitable for patients with HER2-positive advanced UC, offering a new therapeutic option for this population. In contrast, EV plus pembrolizumab is not restricted to HER2-positive patients and covers a broader patient population. Notably, both RC48 and EV carry the MMAE payload, and exposure to MMAE may induce the upregulation of multidrug resistance transporters in tumor cells, resulting in cross-resistance to subsequent MMAE-based therapies. Therefore, sequential use of the two agents is generally not recommended.

During the maintenance therapy phase, a phase II open-label randomized trial (82) enrolled 111 patients with UC whose disease had not progressed following first-line platinum-based chemotherapy. Patients were randomized in a 2:1 ratio to receive sacituzumab govitecan (SG) plus avelumab or avelumab monotherapy. Data from the monotherapy group were adjusted using propensity score weighting, with the JAVELIN Bladder 100 trial serving as an external comparator. The median follow-up durations were 10.94 months and 19.25 months in the combination and monotherapy groups, respectively. The mPFS in the combination group was 11.17 months, significantly longer than 3.75 months in the monotherapy group (HR = 0.49), while the ORR was 24.3% compared with 2.7% in the monotherapy group. However, this combination regimen was associated with substantial toxicity, with grade ≥3 TRAEs occurring in 69.9% of patients; the primary dose-limiting toxicity was neutropenia, and more than half of patients required dose reduction of SG. At the time of reporting, no clear OS benefit had been demonstrated. Chemotherapy-induced bone marrow suppression may compromise hematopoietic and immune function, which, when combined with the myelosuppressive effects of SG, may further exacerbate hematologic toxicity. Overall, while this regimen demonstrates short-term disease control, its risk–benefit profile warrants further optimization and careful clinical evaluation.

During the perioperative period, a phase III randomized controlled trial (83) enrolled 344 patients with muscle-invasive bladder cancer (MIBC) who were intolerant of or refused cisplatin-based regimens. Patients were randomized to receive neoadjuvant EV plus pembrolizumab followed by surgery or surgery alone. With a median follow-up of 25.6 months, the 2-year event-free survival (EFS) rate in the combination group was 74.7% (surgery alone group 39.4%; HR = 0.40, P < 0.001), and the 2-year OS rate was 79.7% (surgery alone group 63.1%; HR = 0.50, P < 0.001); the pathological complete response (pCR) rate reached 57.1%, significantly higher than the 8.6% observed in the surgery alone group. This high pathological response rate provides evidence supporting a bladder-preserving therapeutic strategy. However, all patients receiving combination therapy experienced TRAEs, with grade ≥3 AEs occurring in 71.3% of cases; management of toxicity throughout the perioperative period remains a major clinical challenge. This neoadjuvant regimen significantly improves outcomes in MIBC patients ineligible for cisplatin; however, its toxicity profile limits widespread adoption. Future studies may explore stratified treatment strategies based on circulating tumor DNA (ctDNA) and immune molecular subtypes.

### 4.2 Breast cancer

BC represents a major area of investigation for ADC–ICI combination therapy, with marked differences in clinical benefit observed across distinct molecular subtypes and biomarker-defined populations. Precisely identifying the populations most likely to benefit is therefore a critical prerequisite for successful implementation of combination regimens in this tumor type.

TNBC is the most immunogenic subtype of BC and is also an area where combination therapy with ADCs and ICIs has yielded significant results. A phase III randomized controlled trial (84) enrolled 443 treatment-naïve patients with PD-L1–positive, locally advanced unresectable or metastatic TNBC, who were randomized to receive SG plus pembrolizumab or platinum-based chemotherapy plus pembrolizumab. With a median follow-up of 14.0 months, the mPFS was 11.2 months in the combination therapy group versus 7.8 months in the chemotherapy group (HR = 0.65, P < 0.001). The median duration of response (mDoR) was 16.5 months versus 9.2 months; OS data were not yet mature. The incidence of grade ≥3 TRAEs was 67% in the combination therapy group and 64% in the chemotherapy group; however, the treatment discontinuation rate was lower in the combination therapy group (12% vs. 31%). This difference may be related to the fact that the toxicity profile of the combination therapy regimen is easier to control, as its toxicity can be effectively managed through dose adjustments and symptomatic supportive care; it may also be related to the superior antitumor efficacy observed in the combination therapy group, which can improve patient treatment adherence. This phase III trial provides evidence that ADC–ICI combination therapy may offer advantages over conventional immunochemotherapy in the first-line treatment of PD-L1–positive advanced TNBC. However, since this study compared ADC–ICI with chemotherapy plus ICI—and both groups included an ICI—it is not yet possible to independently assess the additional contribution of the ICI to the efficacy of the ADC based on these results, nor is it sufficient to prove a true synergistic effect between the two; the improved efficacy in the combination group may reflect an additive effect resulting from the different mechanisms of action of the ADC and the ICI. This logic is similar to observations in UC: the EV-302 study confirmed that EV plus pembrolizumab was superior to platinum-based chemotherapy alone, whereas EV monotherapy had already been shown to be superior to chemotherapy in previous studies. This suggests that the benefit of the combination regimen may stem in part from the ADC’s inherently stronger antitumor activity compared with chemotherapy, rather than solely from synergistic gains with the ICI. Whether the PFS advantage of SG plus pembrolizumab in TNBC is also partly attributable to the superiority of SG monotherapy over chemotherapy remains unclear, as there is currently a lack of head-to-head comparison data between SG monotherapy and chemotherapy. Furthermore, this study did not include control groups for SG monotherapy or pembrolizumab monotherapy; therefore, it is impossible to distinguish the respective contributions of ADC monotherapy, ICI monotherapy, and their synergistic effects to the benefit of the combination regimen. Compared with chemotherapy, SG-induced ICD may participate in the regulation of the tumor immune microenvironment by promoting tumor antigen release and immune activation; however, there is currently a lack of direct evidence demonstrating that SG has a stronger effect than conventional chemotherapy in reshaping the TME, and its specific mechanisms of action require further validation.

Unlike TNBC, HR+/HER2− BC generally exhibits an immunosuppressive TME, and whether ADC–ICI combination therapy provides clinical benefit in this subtype remains controversial. A phase II randomized trial (85) enrolled 104 patients with HR+/HER2- metastatic BC. Patients were randomized to receive SG plus pembrolizumab or SG monotherapy. With a median follow-up of 15.5 months, the mPFS was numerically longer in the combination group than in the monotherapy group (8.4 vs. 6.7 months), but the difference did not reach statistical significance (HR = 0.76, one-sided P = 0.12). In the PD-L1–positive (combined positive score [CPS] ≥ 1) subgroup, the mPFS reached 11.1 vs. 5.6 months (HR = 0.51), and the mOS was 18.5 vs. 12.5 months (HR = 0.59), suggesting a potential clinical benefit. Grade ≥3 TRAEs occurred in 71.2% of patients in the combination arm versus 67.3% in the SG monotherapy arm. This pattern of a negative overall result with a positive subgroup signal suggests that in the immunosuppressive microenvironment of HR+/HER2−BC, the overall population is unlikely to derive universal benefit from ADC–ICI combination therapy; however, a benefit signal can be observed in the PD-L1–positive subgroup, indicating that immunomarker-based stratification represents a critical prerequisite for achieving benefit with combination therapy in this subtype. Nevertheless, given the small sample size inherent in this phase II trial, the subgroup findings require further validation in large-scale phase III studies and do not yet support broad clinical application.

In addition, HER2-low BC has recently been established as a distinct therapeutic subtype, and clinical trials evaluating ADC–ICI combination therapy in this population are ongoing. In one treatment arm of a multicenter, open-label, phase Ib/II platform trial (86), 58 patients with previously untreated, locally advanced unresectable or metastatic HR−, HER2-low BC received first-line durvalumab plus trastuzumab deruxtecan (T-DXd). At a median follow-up of 20.6 months, the confirmed ORR was 62.1%, which did not meet the prespecified efficacy threshold of 66.6%. However, the mPFS of 12.6 months and mOS of 30.3 months remain clinically meaningful. Regarding safety, the incidence of drug-related interstitial lung disease (ILD) or pneumonitis was 20.7%, higher than historically reported rates for T-DXd monotherapy (10%–15%), although no increase in grade ≥3 ILD was observed. Although the combination did not meet the prespecified efficacy endpoint, the PFS and OS results suggest potential activity of HER2-targeted ADC–ICI combinations in the HER2-low population. Nevertheless, this regimen may further increase the risk of ILD, underscoring the need to balance efficacy and toxicity in future studies.

### 4.3 Non-small cell lung cancer

Significant progress has also been made in non-small cell lung cancer (NSCLC) with ADC–ICI combination therapy, but the patterns of benefit vary depending on target selection and combination strategy.

TROP2 is widely and highly expressed in NSCLC and represents one of the most actively investigated targets for ADC–ICI combinations. An open-label, multicohort phase Ib trial (87) enrolled 142 patients with driver gene–negative advanced NSCLC to evaluate first-line datopotamab deruxtecan (Dato-DXd) plus pembrolizumab (dual-agent regimen) or pembrolizumab plus platinum-based chemotherapy (triplet regimen). With a median follow-up of approximately 26 months, in treatment-naïve patients, the ORR in the dual-agent cohort (n = 42) was 54.8%, with an mPFS of 11.2 months; mOS had not yet been reached. In the triplet cohort (n = 54), the ORR was 55.6%, mPFS was 6.8 months, and mOS was 17.4 months. Activity was observed across both PD-L1–low and PD-L1–high subgroups. Regarding safety, grade ≥3 TRAEs occurred in 37.1% of patients in the dual-agent group versus 59.7% in the triplet group, with no treatment-related deaths reported. While ORR was comparable between the two regimens, the dual-agent strategy demonstrated a more favorable safety profile. This risk–benefit profile warrants further validation in subsequent trials and provides preliminary evidence supporting the exploration of chemotherapy-free ADC–ICI regimens in advanced NSCLC.

The combination of a TROP2-targeted ADC and ICIs was further validated in a phase III trial. An open-label, randomized phase III study (88) enrolled 413 patients with PD-L1–positive, driver gene–negative advanced NSCLC, who were randomized to receive sacituzumab tirumotecan plus pembrolizumab or pembrolizumab monotherapy. After a median follow-up of 10.5 months, mPFS was not reached in the combination group versus 5.7 months in the monotherapy group (HR = 0.35), with a 12-month PFS rate of 62% versus 29%, respectively. The ORR was 70% in the combination group compared with 42% in the monotherapy group. Grade ≥3 TRAEs occurred in 55% of patients receiving the combination versus 31% receiving monotherapy. This study represents one of the first phase III trials demonstrating that a TROP2-targeted ADC combined with a PD-1 inhibitor significantly improves outcomes compared with PD-1 monotherapy in first-line treatment of PD-L1–positive advanced NSCLC, supporting the clinical development of ADC–ICI combination strategies in this population. However, OS data remain immature, and combination therapy is associated with a higher incidence of adverse events; thus, a careful balance between efficacy and toxicity is required.

In addition, research is underway on the combination of CD30-targeted ADCs with ICIs. A multicohort, open-label phase II trial (89) enrolled 113 patients with advanced NSCLC (n = 55) and cutaneous melanoma (n = 58) who had progressed after prior treatment with PD-1 inhibitors, evaluating the efficacy of brentuximab vedotin (BV) plus pembrolizumab in the post–PD-1 inhibitor resistance setting. In the NSCLC cohort, the ORR was 8% in the primary resistance subgroup, with an mPFS of 2.78 months and a median OS of 10.6 months; in the acquired resistance subgroup, the ORR was 14%, with an mPFS of 5.85 months and a median OS of 14.4 months. Grade ≥3 TRAEs occurred in 31% of patients. Although the absolute efficacy was modest, exploratory biomarker analyses suggested that BV may remodel the TME and potentially overcome resistance to PD-1 inhibitors by selectively depleting CD30+ Tregs. This study represents one of the first clinical evaluations of a CD30-targeted ADC combined with a PD-1 inhibitor in the post–immunotherapy resistance setting, providing mechanistic and preliminary clinical evidence supporting ADC–ICI combinations in resistant solid tumors.

### 4.4 Gastric cancer

Gastric cancer (GC) is one of the key areas of investigation for ADC–ICI combination therapy. Current evidence primarily focuses on later-line treatment in patients with HER2 overexpression.

An open-label, multicenter phase I trial (90) enrolled 56 previously treated patients with HER2-positive advanced GC and other solid tumors to evaluate the safety and preliminary efficacy of disitamab vedotin (RC48) plus toripalimab. Among evaluable patients with GC (n = 28), the ORR was 43%; in the subgroup receiving the recommended phase II dose (RP2D; RC48 2.5 mg/kg) (n = 22), the ORR reached 50%, with an mPFS of 5.1 months and an mOS of 14.0 months. Regarding safety, grade ≥3 TRAEs occurred in 59% of patients. Notably, four patients developed treatment-related pneumonitis; however, no pulmonary toxicity had been observed in historical data for RC48 monotherapy, suggesting a potential increase in immune-mediated pulmonary toxicity associated with ICI combination therapy. A comparison with clinical data on RC48 in UC reveals that pneumonitis was not reported as a prominent safety signal in first-line combination therapy for UC. This suggests that the pneumonitis signal associated with RC48 combined with an ICI may vary across different tumor types and treatment contexts. This discrepancy may be related to prior treatment history. Patients with GC may have received more lines of systemic therapy, whereas patients in first-line combination studies for UC have relatively less prior exposure to systemic therapy. Additionally, factors such as ICI exposure, number of treatment lines, and baseline patient characteristics may also play a role. Further assessment based on prospective safety data across different clinical settings is still required.

At the real-world evidence level, a multicenter retrospective study (n = 57) further validated the efficacy of RC48 plus a PD-1 inhibitor in later-line treatment of HER2-overexpressing advanced GC (91). The combination arm achieved an ORR of 41.7%, significantly higher than 9.5% observed with RC48 monotherapy (P = 0.011), with an mOS of 13.2 months versus 7.1 months for monotherapy (P = 0.040). These real-world findings are consistent with and complementary to the aforementioned clinical trial results, supporting RC48 plus PD-1 inhibitor as a potential later-line treatment strategy for HER2-overexpressing advanced GC. However, given the small sample size, the robustness of the observed efficacy benefit and the long-term safety profile of this regimen require further validation in large-scale prospective clinical trials.

### 4.5 Other solid tumors

As research continues to advance, clinical trials of ADC–ICI combinations are being increasingly conducted across a growing range of solid tumors.

An open-label, multicohort, phase II basket trial (92) enrolled 41 patients with head and neck squamous cell carcinoma (HNSCC) who had not received prior systemic therapy for recurrent/metastatic (R/M) disease, to evaluate the efficacy and safety of EV plus pembrolizumab as first-line treatment for R/M HNSCC. At a median follow-up of 11.0 months, the ORR was 39.0%, with a CR rate of 9.8%, and mPFS was 5.1 months. Subgroup analysis revealed that patients with p16-positive oropharyngeal squamous cell carcinoma achieved an ORR as high as 81.8%. Grade ≥3 TRAEs occurred in 41.5% of patients. The safety profile was consistent with previously reported data for EV plus pembrolizumab, and no new safety signals were identified. This study is among the first clinical trials to evaluate a Nectin-4–targeting ADC combined with a PD-1 inhibitor in the first-line treatment of R/M HNSCC, providing proof-of-concept evidence for this combination strategy. However, the single-arm design and small sample size limit a full assessment of its clinical value.

A phase II, single-arm trial (93) enrolled 18 patients with uterine serous carcinoma (USC) expressing FRα in ≥50% of tumor cells who received mirvetuximab soravtansine (MIRV) plus pembrolizumab. The study demonstrated an ORR of 28%, including one CR and four partial responses (PR), and the 6-month PFS rate was 24.4%. All five responses were observed in patients with no prior exposure to immunotherapy. The incidence of grade ≥3 TRAEs was low, and the regimen was generally well tolerated. However, the study was terminated early due to feasibility constraints in patient enrollment, and the findings require validation in a larger cohort.

Overall, across different cancer types, the efficacy and safety of ADC–ICI combinations do not follow a uniform pattern. On the one hand, some studies show that combining an ADC with an ICI can further improve disease control beyond ICI or chemotherapy alone; on the other hand, limited efficacy, failure to meet the primary endpoint, or increased specific toxicities have also been observed across different tumor subtypes, treatment settings, and combination regimens. These findings suggest that the clinical benefit of ADC–ICI combinations is highly dependent on disease context and treatment setting and may be influenced by factors such as ADC target and payload, tumor immune microenvironment, prior treatment, and combination regimen design.

## 5 Emerging strategies for combination therapy

The previous section primarily focused on the combination of conventional cytotoxic ADCs with ICIs. As ADC payload types, drug structures, and immunotherapeutic modalities continue to expand, the scope of ADC–immunotherapy combinations has also broadened. This section focuses primarily on ADC strategies that go beyond conventional payloads, as well as immunotherapeutic approaches beyond ICIs. By discussing emerging strategies such as ADCs combined with immune agonists and cell therapies, as well as immunostimulatory dual-payload ADCs, and by comparing the mechanisms of action, potential advantages, and limitations of different combination modalities, this section analyzes their potential therapeutic settings and provides a reference for optimizing ADC–immunotherapy combination strategies.

### 5.1 ADCs combined with immune agonists

Immune agonists encompass diverse immunotherapies that can either activate intracellular pattern recognition receptors via small molecules or bind to co-stimulatory receptors on immune cell surfaces via antibodies/ligands, delivering positive activation signals (94). Multiple studies have demonstrated the significant potential of combining ADCs with immune agonists. Rios-Doria et al. (95) showed that ADCs carrying pyrrolobenzodiazepine (PBD) or tubulysin payloads can induce ICD and remodel the TME. In colorectal cancer, renal cell carcinoma, and fibrosarcoma models, these ADCs combined with OX40 or GITR agonist fusion proteins exhibited synergistic antitumor activity. Mechanistically, the combination significantly increased intratumoral CD8^+^ T-cell infiltration and reduced Tregs, thereby alleviating local immune suppression. Notably, this synergistic effect can be achieved at suboptimal ADC doses, enabling potent tumor regression while substantially reducing the off-target systemic toxicity associated with high-dose ADC administration, thereby broadening the therapeutic window. Pascual-Pasto et al. (96) further corroborated this strategy in a mouse model of neuroblastoma, demonstrating that a PBD-based glypican-targeting ADC in combination with a CD40 agonist antibody achieved complete tumor regression in all treated mice, which was durable. Immunophenotypic analysis revealed that the combination therapy markedly increased intratumoral activated CD8^+^ T cells and M1-TAMs, thereby effectively remodeling the TME. Moreover, cured mice developed long-term immune memory.

### 5.2 Immunostimulatory dual-payload ADCs

Immunostimulatory dual-payload ADCs represent a novel class of antibody–drug conjugates in which cytotoxic payloads and immunostimulatory agonists are simultaneously conjugated to the same targeting antibody, enabling a single molecule to mediate both direct tumor killing and *in situ* activation of antitumor immunity (15). Yue et al. (97) constructed a HER2-targeting dual-payload ADC by concurrently conjugating the topoisomerase I inhibitor DXd and the Toll-like receptor 7 (TLR7) agonist MTL1 to a HER2-targeting antibody. Following HER2-mediated internalization, this dual-payload ADC exerts a bifunctional mechanism: on the one hand, release of DXd induces DNA damage, apoptosis, and ICD; on the other hand, intracellular release of the TLR7 agonist activates DCs and macrophages, promoting robust pro-inflammatory cytokine secretion and markedly enhancing intratumoral Teff infiltration. In a trastuzumab-resistant BC model, Tras-DXd-MTL1 demonstrated significantly superior antitumor efficacy compared with single-payload controls (T-DXd or Tras-MTL1) and induced durable antitumor immune memory while effectively suppressing tumor recurrence. Zhou et al. (98) developed a CD276-targeted dual-payload ADC, in which the microtubule-disrupting agent monomethyl auristatin F (MMAF) and the TLR7/8 agonist imiquimod (IMQ) were site-specifically conjugated to the same antibody. This dual-payload construct demonstrated synergistic antitumor activity in a triple-negative BC model, directly inhibiting tumor proliferation while reshaping the TME via TLR7/8 signaling pathways, significantly increasing CD8^+^ T-cell and NK-cell infiltration, and achieving marked tumor burden reduction even as monotherapy. Single-cell RNA sequencing further confirmed that immune cell abundance within tumor tissues was substantially increased following treatment, whereas immunosuppressive pathways were downregulated, resulting in a reversal of the “immune-desert” phenotype.

### 5.3 ADC combined with cell therapy

Cell therapy represents an emerging frontier in the immunotherapy of solid tumors. By genetically engineering and expanding autologous immune cells ex vivo followed by reinfusion into patients, it can elicit durable antitumor immune responses and holds intrinsic potential for synergistic combination with ADCs (99). Jiang et al. (100) generated humanized trophoblast glycoprotein 5T4 (5T4)-targeted antibodies and further constructed ADCs conjugated with DM4 or MMAE payloads, alongside third-generation chimeric antigen receptor T (CAR-T) cells incorporating dual CD28/4-1BB co-stimulatory domains. *In vitro* experiments confirmed that these CAR-T cells specifically recognize 5T4-positive tumor cells and exert potent cytotoxic effects through robust secretion of interferon gamma (IFN-γ) and interleukin-2 (IL-2). In a pancreatic cancer xenograft model, ADC monotherapy achieved sustained tumor growth inhibition with a favorable safety profile. Because both modalities share the same target antigen, they provide a strong rationale for complementary and synergistic combination therapy. In this context, ADCs directly eliminate tumor cells and induce ICD, thereby releasing tumor antigens and remodeling a pro-inflammatory TME, which facilitates CAR-T cell infiltration and activation. Conversely, CAR-T cells provide long-term immune surveillance and eliminate minimal residual disease that cannot be fully eradicated by ADCs. Together, they generate a synergistic effect characterized by “*in situ* immune activation plus long-term tumor clearance”.

Overall, different combination regimens of ADCs and immunotherapy address distinct therapeutic bottlenecks and have varying potential applications. As the regimen with the most robust clinical evidence to date, the combination of ADCs and ICIs primarily relies on ADCs to induce ICD and upregulate PD-L1 expression on tumor cells, thereby reactivating pre-existing endogenous T-cell responses within the tumor. This approach is more suitable for “hot tumors” with a certain degree of T-cell infiltration; however, it has limited efficacy against immune-desert tumors that lack T cells and carries the risk of cumulative organ toxicity from ADCs combined with immune-related adverse events. The combination of ADCs with immune agonists primarily amplifies the ADC-induced antitumor immune response by enhancing innate immune activation or co-stimulatory signaling. Therefore, it is more suitable for tumor types where immune activation is insufficient following antigen release and the innate immune response is relatively weak. However, immune agonists may cause excessive immune activation and inflammation-related toxicity, and their dosage and administration regimens require further optimization. Immunostimulatory dual-payload ADCs integrate a cytotoxic payload with an immunostimulant within a single targeted molecule, enabling the simultaneous induction of cytotoxic effects and immune activation within target cells. Theoretically, they are better suited for scenarios requiring simultaneous enhancement of tumor cell killing and local immune activation; however, their complex molecular structure, payload ratio, and release kinetics may increase the difficulty of drug development and safety control. ADC-cell therapy utilizes ADC-mediated ICD and modulation of the TME to create conditions conducive to the infiltration and activation of effector immune cells, and employs cell therapy to continuously eliminate residual tumor cells. This approach holds unique potential for enhancing immune cell infiltration or controlling minimal residual disease; however, cell therapy still faces challenges such as insufficient infiltration into solid tumors, limited persistence *in vivo*, and associated safety risks. Therefore, the optimal ADC–immunotherapy combination regimen can be selected on an individualized basis based on the characteristics of the ADC target and payload, the state of the tumor immune microenvironment, and the patient’s prior treatment history.

## 6 Current challenges and future prospects

### 6.1 Tumor heterogeneity and bidirectional drug resistance

Tumor heterogeneity represents a major challenge limiting the long-term efficacy of ADC–immunotherapy combinations, with its impact manifesting at two levels. At the intertumoral level, substantial variability in target antigen expression and tumor growth patterns across different solid tumors leads to marked differences in therapeutic responses to identical combination regimens. At the intratumoral level, tumor cells within the same lesion exhibit heterogeneous differentiation states; while a subset of cells with high antigen expression can be effectively eliminated by ADC-mediated cytotoxicity, a considerable fraction of cells with low antigen expression do not respond effectively, thereby serving as a reservoir for recurrence and metastasis (101, 102). The coexistence of intertumoral and intratumoral heterogeneity contributes to both primary and acquired drug resistance, with these resistance processes mutually reinforcing tumor progression and increasing metastatic potential. Currently, there is a lack of reliable predictive biomarkers for pretreatment resistance stratification, as well as limited long-term intervention strategies, thereby restricting the clinical efficacy of combination regimens.

To address the aforementioned challenges, future efforts should be pursued at three levels. At the research level, efforts should focus on integrating long-term follow-up data, liquid biopsy data, and multicenter cohort datasets to systematically analyze drug resistance evolution across different cancer types and molecular subtypes, and clarify differences in clinical characteristics between patients with primary and acquired resistance. At the drug development level, next-generation ADCs should be iteratively developed, with optimization of linker design and conjugation strategies, reduction of antigen escape windows, and enhanced coverage of tumor heterogeneity. At the clinical level, combination therapy regimens should be expanded to include immune agonists, bispecific molecules, or novel ICIs, thereby delaying the emergence of drug resistance through multi-pathway synergistic effects and achieving long-term tumor control.

### 6.2 Challenges in safety management of combination therapies

When ADCs are combined with immunotherapy, adverse events from both modalities may accumulate, making safety management more complex. Immunotherapy commonly causes adverse events such as immune-mediated pneumonitis, dermatitis, and neurotoxicity (103), while ADCs frequently lead to bone marrow suppression, interstitial lung disease, and peripheral neuropathy (104). Among these, immunotherapy-related pneumonitis and ADC-related ILD overlap in their clinical manifestations; their combined use may increase both the difficulty of identifying pulmonary adverse events and the risk of their occurrence. When used in combination, the incidence of severe adverse events is significantly higher than with monotherapy; these reactions occur earlier, affect a wider range of organs, and often manifest as multiple concurrent toxicities (105). Once severe toxicity occurs, clinicians typically reduce the dose, suspend treatment, or even permanently discontinue the combination regimen. This not only directly interrupts the therapeutic process by which ADCs continuously reshape the tumor immune microenvironment but also weakens the overall antitumor effect. At the same time, persistent adverse reactions severely impact patients’ quality of life, making it difficult for some patients to maintain standard treatment.

To better control safety risks, a standardized management workflow covering the entire treatment course should be established in the future. At baseline, high-risk patients should be systematically screened, and alternative strategies should be developed in advance. During treatment, regular follow-up and monitoring should be conducted, doses should be flexibly adjusted according to toxicity grading, sequential dosing strategies should be explored, and a multidisciplinary team should be assembled to develop individualized regimens, thereby ensuring the continuity of combination therapy. At the same time, patient education should be strengthened to encourage proactive reporting of adverse events and promote early symptom recognition and intervention. In addition, different ADCs and immunotherapies have distinct toxicity profiles. When selecting future combination regimens, in addition to efficacy, the degree of overlap between toxicity profiles should also be considered, with priority given to drug combinations with minimal overlap in toxicity profiles. By optimizing dosing and administration sequences, toxicity can be reduced and the therapeutic window can be expanded.

### 6.3 Challenges in biomarker development and precision medicine

Currently, clinical methods for identifying patients likely to benefit from ADC–immunotherapy combination remain limited, relying primarily on two indicators: tumor target antigen expression levels and PD-L1 status (48, 106). This evaluation framework does not adequately capture tumor heterogeneity or the overall immune status of patients, making it challenging to accurately identify true responders to combination therapy. In addition, validated dynamic biomarkers for treatment monitoring are lacking in clinical practice, limiting real-time assessment of therapeutic response and hindering early prediction of drug resistance or disease progression. This, in turn, restricts the development of personalized treatment strategies and impedes the clinical implementation of precision medicine.

With the rapid development of spatial multi-omics, liquid biopsy, and AI-driven big data analytics, a multi-level evaluation system integrating “baseline stratification and dynamic monitoring” should be established in the future. Prior to treatment, multidimensional assessments should be performed to distinguish patients suitable for monotherapy from those who may benefit from combination therapy. During treatment, liquid biopsy and peripheral blood biomarkers should be used to dynamically monitor immune response intensity and provide early warning of drug resistance. However, circulating biomarkers primarily reflect the peripheral circulation and cannot fully capture the local TME or its spatial heterogeneity. Therefore, liquid biopsy results may deviate from the true immune status of the tumor tissue. Consequently, future efforts should integrate tissue-based markers, spatial multi-omics, and other information for comprehensive interpretation to improve the accuracy of dynamic monitoring. Meanwhile, efforts should be made to standardize biomarker testing procedures and interpretation criteria, enabling objective and consistent results to guide individualized treatment strategies and precisely identify patient subgroups most likely to benefit, thereby improving overall therapeutic outcomes.

### 6.4 Lack of standardization in clinical settings and dosage strategies

Currently, clinical trials of ADC–immunotherapy combinations are distributed across multiple treatment settings, with substantial heterogeneity in drug selection, dosing regimens, dosing intervals, and sequencing strategies; a universally accepted standardized protocol has not yet been established worldwide (107, 108). First, evidence supporting optimal dosing regimens remains limited. In theory, administering ADCs first to remodel the TME, followed by sequential immunotherapy, may more effectively reduce toxicity while enhancing synergistic antitumor effects. However, this hypothesis lacks support from large-scale data. Furthermore, since traditional ADCs and ICIs typically have long *in vivo* half-lives, sequential administration of ADCs followed by immunotherapy may be limited by sustained drug exposure; therefore, concurrent administration remains the primary approach in current clinical practice. Second, evidence in perioperative settings remains insufficient. Combination strategies in the perioperative setting across most cancer types are still underdeveloped, and standardized dose modification protocols for low-risk patients are lacking. Third, standardized stratification guidelines are lacking. TME characteristics and immunological profiles vary significantly across cancer types; however, no consensus guidelines exist for treatment stratification based on tumor type or disease stage.

Future research should focus on three areas: First, the implementation of sequential strategies may require conjugates with shorter half-lives. Short-acting conjugates, such as BDCs and small-molecule drug conjugates (SMDCs), exhibit more rapid clearance *in vivo* (109, 110). Future studies could explore a strategy of first administering a short-acting toxin conjugate, followed by sequential administration of immunotherapy after the conjugate has been cleared from the circulation. Multicenter, stratified controlled trials could also be conducted to determine the optimal dosing regimens and administration sequences for different cancer types and disease stages. Second, early-stage treatment settings such as neoadjuvant and adjuvant therapy should be expanded, and the evidence base for perioperative treatment should be strengthened. Meanwhile, liquid biopsy-based dynamic monitoring of ctDNA could be used to develop individualized dose-reduction or treatment discontinuation strategies for low-risk patients with postoperative minimal residual disease (MRD)-negative status, thereby shortening unnecessary periods of combination therapy. Finally, based on the target antigen types and immune microenvironment characteristics of different tumors, standardized treatment protocols should be established for individual cancer types, and medication guidelines should be refined for specific clinical scenarios, thereby optimizing the combined antitumor efficacy of ADCs and immunotherapy.

## 7 Conclusion

ADC–immunotherapy combinations have demonstrated favorable antitumor effects across multiple tumor types. The underlying mechanisms mainly involve payload-mediated ICD, Fc domain-driven activation of innate immunity, and the cleavable linker-mediated bystander effect, which together remodel the tumor immune microenvironment; in turn, the immune microenvironment can also affect the antitumor activity of ADCs. Clinical evidence indicates that this combination strategy has demonstrated clinical benefit across a range of solid tumors, with a generally manageable safety profile. Emerging combination approaches continue to expand its therapeutic potential. However, the clinical translation of this combined strategy remains constrained by the complexity of tumor biology and the lack of mature clinical management protocols. Future efforts should focus on driving the field toward precision and standardization to fully unlock the synergistic potential of ADCs and immunotherapy, thereby delivering lasting and stable clinical benefits to cancer patients.
